# Context Sensitivity and Previous-Choice Associations in Repeated Shot-Direction Decisions Among Adolescent Badminton Players

**DOI:** 10.3390/bs16101721

**Published:** 2026-09-22

**Authors:** Senlin Lan, Jialian Cao, Jiayi Wu, Hongli Liang, Guanchen Zhou, Zhihua Yang, Zhipeng Chen, Haoping Yang, Ye Mao, Xiaotong Wang, Hui Huang

**Affiliations:** 1 School of Competitive Sports, Beijing Sport University, Beijing 100084, China; 2 School of Physical Education, South China University of Technology, Guangzhou 510641, China; 3 School of Physical Education & Sports Science, South China Normal University, Guangzhou 510006, China; 4 School of Competitive Sports, Huaqiao University, Quanzhou 362021, China; 5 School of Psychology, Beijing Sport University, Beijing 100084, China

**Keywords:** adolescent athletes, badminton, decision-making, context sensitivity, previous choice, sequential choice

## Abstract

This study examined whether repeated shot-direction decisions in adolescent badminton players were associated with the current spatial context and the immediately preceding choice. Fifty systematically trained players aged 13–15 years completed a hidden-probability phase (Task 1), in which no numerical outcome probabilities were displayed at choice, followed by an explicit-probability phase (Task 2), in which one current outcome-probability dimension was displayed for the two route options. Task 1 constituted the primary analysis, and Task 2 provided a secondary boundary-condition assessment of the same choice structure. Primary inference was based on trial-level binomial generalized linear mixed-effects models for Task 1, complemented by rolling forward validation of out-of-sample prediction. In Task 1, relative to the center position, a left opponent position was associated with lower odds of a straight choice (OR = 0.087, 95% CI [0.059, 0.130]), whereas a right position was associated with higher odds (OR = 6.297, 95% CI [4.400, 9.013]). After adjustment for current context, trial progress, and position sequence, a previous straight choice was associated with lower odds of choosing straight on the next trial (OR = 0.430, 95% CI [0.339, 0.546]). Adding previous choice improved out-of-sample predictive performance (ΔELPD = 18.081, 95% CI [5.277, 30.885]), although the absolute improvement was modest. Task 2 produced directionally similar estimates in the mixed model, while participant-clustered generalized estimating equations provided weaker support for this association; this phase was therefore interpreted descriptively. Outcome-dependent and contextual Q-learning models showed no stable incremental predictive support beyond the context-plus-previous-choice baseline. Because route choice, screen location, and keypress were coupled by design, the previous-choice term indexes a composite history signal spanning tactical, spatial, and motor-response components.

## 1. Introduction

In competitive settings, athletes repeatedly choose actions in response to changing perceptual information and spatial relations. During a match, each decision develops within an ongoing perception–action process (Beck et al., 2026). Experimental paradigms typically sample this process through discrete decision trials. Evidence from repeated-choice paradigms shows that responses on a given trial can be systematically associated with preceding stimuli, choices, and responses, even when successive trials are nominally independent (Gao et al., 2009; Fründ et al., 2014; Braun et al., 2018; Urai et al., 2019). Overall choice proportions, mean accuracy, or mean response time may therefore describe the average response tendency while leaving these trial-to-trial associations unexamined. In sport-specific tasks, examining such associations is particularly relevant because current contextual information and recent response history may contribute simultaneously to the observed choice pattern.

Badminton is an open-skill sport in which decision-making is constrained by rapidly changing spatial and temporal information. Shuttle trajectory, player position, stroke preparation, and available court space can change within a single exchange. Badminton anticipation studies show that players use both kinematic and contextual information when judging an opponent’s likely action, with court configuration and preceding rally information contributing to these judgments (Robertson et al., 2022; De Waelle et al., 2023; Lan et al., 2026). Naturalistic research with expert badminton players further indicates that shot selection is informed by the player’s own situation, the opponent, the available court space, and the developing rally; reported tactical intentions include playing wide, applying pressure, and creating surprise (Macquet & Fleurance, 2007). Direct experimental evidence linking opponent position to a player’s own shot-direction selection in badminton remains limited. This unresolved relationship provides the basis for examining opponent position as a contextual predictor of straight and cross-court route choices in the present study.

Accordingly, opponent position was operationalized as a measurable contextual predictor. For overhead attacking situations, the opponent’s position was classified as left, center, or right from the player’s perspective, and participants chose between straight and cross-court routes. Straight and cross-court were treated as alternative response options, with route choice modeled as a function of the current spatial context. This operationalization allowed us first to quantify the association between the current spatial context and route choice and then to test whether the immediately preceding choice contributed additional information beyond that contextual baseline.

Sport cognition research has consistently shown that expertise is associated with advantages in anticipation, cue extraction, visual search, and decision accuracy (Mann et al., 2007; Müller & Abernethy, 2012; Loffing & Cañal-Bruland, 2017). In badminton, these perceptual–cognitive demands vary across stroke situations and depend on the information available before action execution (Robertson et al., 2022; De Waelle et al., 2023; Lan et al., 2026). Adolescence is particularly relevant to the present question because perceptual–cognitive and decision processes continue to develop during a period of rapidly accumulating sport-specific experience. Recent work in adolescent badminton players has identified age- and competitive-level differences in perceptual–cognitive performance, including working-memory-related measures (Wu et al., 2024). Developmental decision-making research further indicates age-related variation in how recent outcomes and experience contribute to subsequent choices (Jepma et al., 2022). Examining trial-to-trial associations in systematically trained adolescent players therefore provides a useful setting for characterizing the contribution of recent response history during a developmental period in which domain-specific experience is already substantial and perceptual–cognitive functions continue to mature.

The present sample combined a relatively narrow age range of 13–15 years with 5–7 years of systematic badminton training. This profile reduced broad developmental heterogeneity while ensuring sustained exposure to badminton-specific spatial relations, stroke options, and competitive situations. Repeated-choice behavior could therefore be examined in athletes with substantial familiarity with the task context within a clearly defined developmental window. The present study characterizes a repeated-choice structure within this adolescent badminton population, with developmental differences across age groups providing a direction for future comparative work.

Two athletes completing the same repeated binary-choice task may show similar overall choice proportions while differing in how their responses unfold across trials. Work on sequential decision-making has shown that current responses can be associated with recent stimuli, choices, and actions, which produces repetition or alternation biases even when successive trials are experimentally independent (Fründ et al., 2014; Urai et al., 2019). More recent studies have reinforced this distinction between current evidence and recent decision history. Feigin et al. (2021), for example, found that prior choices could bias subsequent perceptual decisions even when the motor response used to report those choices changed, whereas recent reviews have emphasized that serial dependence may arise at multiple perceptual, decisional, and response-related stages (Pascucci et al., 2023; Manassi et al., 2023). Trial-history effects also continue to be incorporated into contemporary evidence-accumulation accounts of decision-making (Gupta et al., 2024).

In sport-specific tasks, sequence patterns may arise jointly from recent response history and changes in the current context. If opponent position differs across adjacent trials, context-dependent choice probabilities alone can generate apparent repetition or alternation. Sequential analyses therefore need to account for both recent responses and the current and preceding contexts. Given the fixed mapping among route, screen location, and keypress, previous choice was modeled as an observed lagged predictor indexing their combined tactical, spatial, and motor-response history.

Importantly, the repeated trials in the present task did not represent consecutive actions within the same rally. Each trial was an independently recorded video, and trial order was randomized. The “previous choice” variable therefore refers to the participant’s response on the immediately preceding experimental trial, not to a tactically connected preceding shot in an ongoing rally. Accordingly, this study addresses trial-to-trial choice associations within a repeated sport-specific decision task rather than continuous tactical adaptation during match play.

Observed repetition or alternation establishes a trial-history association without specifying its underlying process. The primary analysis therefore tested whether the immediately preceding response remained associated with the current decision after accounting for current and preceding spatial context. We complemented this inferential analysis with rolling out-of-sample prediction to assess whether recent-response information improved prediction beyond context alone (Yarkoni & Westfall, 2017).

We subsequently evaluated whether recent outcome information or gradual action-value updating contributed additional predictive value beyond the context-plus-previous-choice baseline. These analyses were treated as comparisons among candidate predictive structures, with detailed model definitions, estimation procedures, and validation settings reported in the Methods (Palminteri et al., 2017; Wilson & Collins, 2019).

Adjacent responses were drawn from independently recorded videos presented in randomized order, with the experimental trial sequence defined as the relevant level of analysis. Given the limited prior evidence on sequential shot-direction choice in adolescent badminton players, this study was framed around three exploratory, non-directional research questions. First, was the opponent’s current position associated with the probability of choosing a straight or cross-court route? Second, after accounting for the current and preceding spatial context, was the immediately preceding choice associated with the current decision, and did it improve prediction in later trials? Third, beyond the context-plus-previous-choice baseline, did outcome-sensitive or value-updating structures provide additional out-of-sample predictive information? Task 1 provided the primary test of context sensitivity and previous-choice association under conditions in which numerical outcome probabilities were unavailable at the moment of choice. Task 2 extended the same decision structure by introducing explicit current outcome information while retaining the sport-specific visual context and response format. Its purpose was to examine a boundary condition of the primary finding: whether associations with current context and recent response history remained observable when participants could also consult externally provided outcome information. Task 2 followed Task 1 and reused the same stimuli; as such, it was positioned as a secondary within-paradigm extension. It characterized the choice structure under an information-enriched decision setting, with cross-phase differences serving a descriptive role.

## 2. Materials and Methods

### 2.1. Participants

The sample comprised 50 adolescent badminton players (36 boys and 14 girls). On-site registration records showed that participants were 13–15 years old (M = 13.70, SD = 0.71): 22 were aged 13, 21 were aged 14, and 7 were aged 15. Recruitment focused on junior-secondary-school athletes to provide a relatively homogeneous developmental window, with systematic training ensuring sustained experience of badminton-specific perceptual and tactical situations. They had received systematic badminton training for 5–7 years (M = 5.82, SD = 0.80): 21 had trained for 5 years, 17 for 6 years, and 12 for 7 years. Age, sex, and training duration were recorded before the experiment. This exploratory study was conducted without a formal a priori power calculation. Recruitment from the collaborating training setting yielded 50 eligible participants, each scheduled to complete 90 Task 1 trials. The repeated-trial design provided dense within-participant information for estimation of the prespecified trial-level associations. Sample precision was evaluated from the confidence interval of the primary population-level previous-choice coefficient, while the 50 independent participants defined the information available for estimating between-participant heterogeneity. The final Task 1 dataset comprised 4402 valid binary choices and 4295 valid adjacent transitions; the primary previous-choice estimate was OR = 0.430 with a 95% CI of [0.339, 0.546]. The participant-level sample provides more limited information for the highly parameterized computational models and individual-difference estimates. Primary inference therefore centered on the trial-level GLMM, with the computational models treated as secondary analyses and evaluated through held-out prediction, sensitivity analyses, and parameter-stability diagnostics. Participants were recruited through purposive convenience sampling from the Focus Badminton Club in Guangzhou. Eligible participants met the predefined age and systematic-training criteria within the collaborating training setting. All participants attended junior secondary school, had sustained badminton training and competition experience, and were registered members of the Nansha District badminton representative team in Guangzhou; none had obtained a formal Chinese athlete technical-grade certificate at the time of testing. The resulting cohort comprised systematically trained adolescent badminton players from this training environment.

Before testing, the researchers separately explained the study purpose, procedures, possible burden, and right to withdraw to the guardians and adolescents. Guardians provided written informed consent, and the adolescents provided corresponding written assent. The project supporting this study was reviewed and approved by the Sports Science Experiment Ethics Committee of Beijing Sport University (approval No. 2025376H; 18 September 2025). Participant codes were used to de-identify the data during analysis and reduce direct linkage between personal identifiers and behavioral records.

### 2.2. Task Design and Experimental Paradigm

We used a repeated video-based route-choice paradigm based on an overhead badminton smash situation. It comprised a hidden-probability phase (Task 1) followed in a fixed order by an explicit-probability phase (Task 2). The design needed to retain sport-specific context while allowing repeatable coding of the current spatial context and route choice. We therefore restricted the target scene to relatively homogeneous rear-court overhead attacks; represented the current spatial context by the opponent’s left, center, or right position from the player’s perspective; and treated straight and cross-court routes following the same attacking action as a binary choice. These two routes do not exhaust the tactical options available in actual matches, and the design did not assume a uniquely optimal route at any position. The two phases shared the same stimulus structure, choice method, and outcome-feedback framework. Their central difference was that the Task 2 choice screen additionally displayed probabilities for one outcome dimension. Because Task 2 always followed Task 1 and reused the stimuli, the phases were sequential components of one paradigm rather than independent tasks.

Video stimuli were generated from rallies performed by six male national first-class badminton players, each with more than 15 years of systematic training experience. The exchanges preceding the target stroke were played as genuine rallies, allowing the players to select their shots in response to the shuttle trajectory and court situation. Clips could begin at the serve or at a later stroke, which thereby preserved variation in the contextual development preceding the target action. The formal stimulus pool was stratified by opponent position and the route originally taken in the video, with approximately 50% straight and 50% cross-court clips. These two variables constituted the formal stratification factors. Clips involving all six players were interleaved in randomized order during task presentation. Preceding rally length was retained as natural stimulus variation within the standardized target configuration; all retained clips contained at least four effective preceding strokes and converged on the same rear-court attacking situation. This organization limited systematic clustering of individual player characteristics while preserving variation in rally development across trials. Filming took place in the gymnasium at Beijing Sport University under quiet, distraction-free conditions. Of the 150 candidate videos initially recorded, 120 were retained after screening against the five criteria described below. Two coauthors conducted the screening. Independent coder-level screening decisions were not retained, precluding retrospective quantification of inter-rater agreement for stimulus inclusion. This limits the assessment of reliability for the subjective elements of the video-screening procedure.

Experimental control was imposed mainly on the formation of the target situation. The players first completed the preceding exchange normally. Rally length was allowed to vary across stimuli, with standardization focused on the formation of the target attacking situation. The players completed the preceding exchange naturally until the far-court player returned the shuttle to the near-court player’s overhead area, producing the intended rear-court attacking opportunity. Candidate videos with fewer than four effective preceding strokes were excluded to ensure that sufficient contextual information was available. The target incoming location and attacking configuration therefore constituted the principal controlled features across stimuli. Apart from this target incoming location, the preceding route organization and stroke choices remained responsive to the actual rally. This procedure preserved the natural development of the preceding exchange, including shuttle trajectories and spatial relations, while standardizing the final target situation across videos.

Candidate videos were screened for a complete and usable target situation. Videos were excluded when (1) the target return landed too shallow, inside the long doubles service line, producing an obvious mid-court ball rather than a rear-court overhead attack; (2) fewer than four effective rally strokes preceded the target action, leaving insufficient contextual information; (3) the hitter was clearly leaning backward, off balance, or otherwise substantially constrained at the target stroke; (4) the final route was already evident from the action before occlusion; or (5) the opponent had moved almost entirely to one side and had effectively abandoned the other side, creating an excessively strong and near-deterministic spatial cue. Screening reduced cases in which imbalance, extreme positioning, or early route disclosure directly determined the choice and retained sport-specific decisions with meaningful uncertainty.

Temporal occlusion occurred after the target action had formed but before the final route was revealed by clear racket movement. Each video ended when the hitter had completed trunk arching and assumed an overhead smash preparation posture with the upper limb in a ready position but before an evident backswing and accelerated forward swing could disclose the route. The route-choice screen appeared immediately. Participants could use the preceding rally, opponent position, shuttle trajectory, and the hitter’s early posture while being prevented from seeing the final racket motion and actual shot direction. This treatment is consistent with pre-contact temporal occlusion in badminton anticipation studies (De Waelle et al., 2023; Lan et al., 2026).

Stimuli were filmed from the near-court player’s rear-court perspective and showed the near-court hitter, the net, and the far-court opponent. Each clip retained the exchanges preceding the target stroke to provide information about action development and spatial relations; trials were otherwise independent videos. Independent clips permitted repeatable trial sequences and separate trial-level modeling of current position and previous choice, without equating a naturally continuing tactical state within one rally with intertrial dependence. The formal pool contained 120 MP4 videos arranged in six strata defined by three opponent positions and two original video routes, with 20 videos per stratum. For Task 1, each participant received 90 videos drawn without replacement and organized into three blocks of 30 trials. Each block included 10 trials at each opponent position and, within each position, five originally straight and five originally cross-court videos. Task 2 reused the videos shown to that participant in Task 1 rather than drawing an independent set. Among the 47 participants who completed 90 trials in both phases, 46 received exactly the same video set and order in both phases; one received the same 90 videos in a different order. Available trials from the three incomplete task records also overlapped with the corresponding stimuli in the other phase. Inventory records indicated 1920 × 1080 resolution, 60 fps, and a duration of 6–8 s (M = 6.789 s); audio was disabled. After stratified quota sampling, the 30 trials within each block were randomized, and every participant received an independently generated random sequence.

Opponent position was coded as left, center, or right from the near-court player’s perspective and served as the current trial’s sport-specific spatial context; no route was presumed objectively optimal. The response screen used a fixed spatial–key mapping: straight always appeared on the left and was selected with the F key, whereas cross-court always appeared on the right and was selected with the J key. All participants responded with the right hand.

### 2.3. Construction of Expert-Derived Probabilities

A controlled, non-deterministic feedback environment was constructed from sport-experience judgments, yielding three-category outcome probabilities for each opponent-position × route-choice condition. These probabilities governed the WIN, LOSS, and RALLY feedback presented during the task. It established a controlled probabilistic environment rather than estimating objective route success in actual competition. Judgments were obtained from 26 raters with substantial badminton-specific experience. Eligibility required at least six years of badminton training together with either a formal athlete technical grade or experience coaching adolescent badminton players. The panel combined substantial domain-specific experience with variation in competitive grade and coaching background. The resulting probabilities summarize the aggregated judgments of this expert group. The final sample included one national master-level athlete, 19 first-class athletes, five second-class athletes, and one person without a formal grade; training experience ranged from 6 to 18 years (M = 13.27). Twenty-two had coached adolescent badminton players, and four had not.

Experts rated the six conditions formed by three opponent positions and two attacking routes. For each condition, they estimated the probabilities of WIN (point won), LOSS (point lost), and RALLY (rally continues). Response options ranged from 0% to 100% in 10-percentage-point increments. Exported data contained option codes, which were converted back to percentages before analysis. When the three probabilities within a position–route condition did not sum to 100%, that condition was treated as invalid and excluded from aggregation. Across the 156 possible rater × condition probability sets, 17 were excluded under this criterion, which left 139 valid sets. The exclusions involved six raters and were distributed across all six position–route conditions. Condition-level valid sample sizes ranged from 21 to 24, with the largest number of invalid sets observed for the left–straight condition (5 of 26).

For each condition, valid judgments were first averaged by outcome dimension. The three mean values were then normalized within the condition so that their sum equaled 100%, and the normalized values were rounded to integers by the largest-remainder method. The resulting integer probabilities formed the feedback-sampling matrix. In Task 1 and Task 2, outcome feedback was sampled from this matrix after each valid choice. Of the 26 raters, 20 provided valid judgments for all six conditions. Their ratings were used to estimate average-measure absolute agreement using ICC(A,k). The ICC values were 0.918 (95% CI [0.79, 0.99]) for WIN, 0.894 (95% CI [0.73, 0.98]) for LOSS, and 0.776 (95% CI [0.47, 0.96]) for RALLY. The RALLY estimate showed lower precision than the two terminal outcome categories. A sensitivity analysis comparing all valid condition-level ratings with the fixed subset of 20 complete raters yielded a maximum absolute difference of 1.83 percentage points across the 18 normalized cells, with all differences below 2 percentage points. The implemented feedback matrix was therefore stable across the two inclusion rules despite greater uncertainty in the RALLY ratings. These values functioned as expert-derived parameters of the experimental environment and were used to generate task feedback.

### 2.4. Experimental Procedure

All participants completed Task 1 and then Task 2 before their regular training. The phases were administered in a fixed order. Apart from adding one displayed outcome-probability dimension to the choice screen, Task 2 reused the video materials already experienced in Task 1; most complete records also retained the same presentation order. Participants therefore entered Task 2 with prior experience of the stimuli, task structure, and feedback environment. This design prevented Task 2 from serving as an independent replication of Task 1 or an unconfounded causal comparison. A start screen appeared before each block, and participants pressed the space bar to continue. Before formal testing, the researcher explained video viewing, route choice, the F/J mapping, and feedback rules. Ten practice trials familiarized participants with stimulus presentation, choice, and feedback and were excluded from analysis. The experiment was implemented in MATLAB R2023a (The MathWorks, Inc., Natick, MA, USA) with Psychtoolbox-3 version 3.0.17 and run on a Lenovo Xiaoxin Pro 16 AHP9 laptop (Lenovo, Beijing, China). Visual stimuli appeared at the center of a 16-inch display with 1920 × 1080 resolution and a 60 Hz refresh rate. Viewing distance was approximately 60 cm. The room was quiet with stable lighting, and responses were recorded on a standard keyboard.

Figure 1 shows the trial sequence. A trial began with a 300 ms black-screen intertrial interval and a 500 ms central fixation point, followed by the complete pre-edited video containing the preceding rally. The video ended at the critical decision moment before racket–shuttle contact, and the binary-choice screen appeared immediately; response timing began when this screen was displayed. Participants had 1500 ms to press F for straight or J for cross-court. After a valid keypress, the selected option was highlighted with a yellow border for 250 ms, followed by 1000 ms of outcome feedback and the cumulative score. The 1500 ms window was selected as a pragmatic standardized deadline to preserve a relatively rapid post-occlusion decision interval while maintaining feasibility for the binary keypress response in adolescent participants. The same deadline was applied across participants and task phases, with response-time availability held constant throughout the experiment. Because this deadline constrained the time available for choice, the present findings characterize decision behavior under this fixed temporal setting. A WIN increased the participant’s score by 1, a LOSS increased the opponent’s score by 1, and a RALLY left the score unchanged. If no response occurred within 1500 ms, a timeout was recorded, no outcome was generated, the score did not change, and the next trial began.

The three opponent-position categories used as the current sport-specific spatial context are illustrated in Figure 2.

The Task 1 choice screen displayed only straight and cross-court and provided no numerical probability for the current trial. Choice was therefore initially based on action and spatial information from the video. After a choice, however, WIN, LOSS, or RALLY was still sampled from the complete distribution for the applicable opponent-position × actual-choice condition in Table 1. Hidden probability refers only to the absence of displayed numerical probabilities at choice; repeated feedback could still support experience-based learning. Agreement estimates for the expert probability ratings are summarized in Table 2.

Task 2 introduced current numerical information into the same stimulus and choice structure. In each trial, one outcome dimension—WIN, LOSS, or RALLY—was displayed together with the corresponding probabilities for straight and cross-court. Each display type occurred 30 times across the 90 trials and no more than three times consecutively. The displayed information did not alter outcome generation, which continued to follow the complete WIN/LOSS/RALLY distribution for the relevant opponent-position × chosen-route condition.

This phase was designed as a secondary boundary-condition analysis of the repeated-choice structure. Specifically, it allowed the context and previous-choice associations to be examined after explicit outcome information became available at the time of choice and permitted adjustment for the information displayed in each trial. Task 2 retained the same videos to preserve the underlying sport-specific situations across phases. Because Task 2 followed Task 1, participants entered this phase with prior stimulus exposure, accumulated feedback experience, and task practice, alongside possible transfer and fatigue. Task 2 was therefore analyzed as an information-enriched descriptive phase, with cross-phase estimates used descriptively.

### 2.5. Data Analysis

The dataset contained 8872 records: 4478 from Task 1 and 4394 from Task 2. Valid binary choices numbered 4402 and 4307, respectively. Current route was coded straight = 1 and cross-court = 0, with center as the reference opponent position. Trial progress was defined as (trial number − 1)/89 and centered at 0.5. The previous-choice variable was created only when trial numbers were consecutive within the same participant and task. An invalid choice on the preceding trial broke the lag; the most recent earlier valid choice was not carried forward across a missing response. Because straight–left screen–F key and cross-court–right screen–J key were fixed mappings, previous route, keypress, and spatial response were perfectly collinear. The lagged variable is referred to as previous choice throughout, with its coefficient indexing the combined history of shot direction, spatial response, and keypress under the fixed mapping. Adjacent-trial analyses included 4295 Task 1 transitions and 4176 Task 2 transitions. The most recent valid choice at the same opponent position was examined separately as a sensitivity variable and was not conflated with the immediately preceding choice.

Data-quality checks found no duplicate participant–task–trial keys and no illegal codes in non-missing position, choice, or outcome fields. Choice and outcome were both missing on 163 of 8872 recorded rows (1.84% overall): 76 of 4478 Task 1 rows (1.70%) and 87 of 4394 Task 2 rows (1.98%). Missing choices did not enter the binomial likelihood and broke construction of the previous-choice variable. In addition to within-trial nonresponses, Task 1 for P008 and Task 2 for P024 and P046 ended early because of program errors. These records contained 68, 39, and 35 trials and 64, 38, and 32 valid binary choices, respectively. Trial-level mixed models can use unequal numbers of observations, so all 50 participants remained in the primary analysis. A sensitivity analysis restricted each phase to participants with at least 80 valid choices to test whether the clearly incomplete records affected the main results.

We first estimated the probability of a straight choice at each opponent position in Tasks 1 and 2, with participant-clustered 95% intervals, and used binomial-logit GLMMs to examine the conditional association between current position and route choice. The Task 1 previous-choice GLMM was the primary inferential model. Position effects denote conditional statistical associations, not causal effects or psychological mechanisms. The context model for position included a participant random intercept and mutually independent random slopes for the left and right contexts. A likelihood-ratio test assessed the Task 1 position × trial-progress interaction. For the Task 1 previous-choice test, two models were compared on the same 4295 valid adjacent transitions with identical random-effects structures. Both included current position, position × trial progress, previous position, same-position transition, a participant random intercept, and mutually independent random slopes for left position, right position, and previous choice. The full model added only the fixed effect of previous choice; the likelihood-ratio test therefore assessed the population-level previous-choice coefficient. Outcome conditioning was an extension after the primary model and added previous outcome and the previous-choice × previous-outcome interaction.

GLMMs were fitted with glmer in R 4.5.2 and lme4 2.0-6 using the bobyqa optimizer and Laplace approximation (nAGQ = 1). The main script used maxfun = 200,000; the explicit-information and previous-choice robustness scripts used maxfun = 300,000. Fixed effects are reported as ORs, Wald 95% CIs, and *p* values; nested models were compared with likelihood-ratio χ^2^ tests. Participant random intercepts and slopes used independent parameterization (||). Candidate random-effects structures were ordered separately for the context and sequential models. All primary models met the convergence criteria and were nonsingular with the first candidate structure, so no simplification was required. Task 2 explicit-information models and video-random-intercept models were fitted directly from their specified formulas. Only the secondary Task 2 same-position sensitivity model required removal of the previous-choice random slope for stable estimation. Convergence checks used lme4 messages, optimizer return codes, and maximum absolute gradients; singularity was assessed with isSingular(tol = 1 × 10^−4^). Complete fixed-effect estimates for the primary GLMMs and the corresponding random-effects structures and fitting diagnostics are reported in Supplementary Tables S1 and S1A. Participant-clustered generalized estimating equations (GEEs) were fitted with geeglm in geepack 1.3.13 using a binomial distribution, logit link, participant clusters, an independence working correlation, and robust sandwich standard errors (std.err = “san.se”). The same GEE implementation was used for both tasks. Five Task 1 robustness analyses examined the sensitivity of the previous-choice coefficient to context coding, stimulus structure, estimation framework, and incomplete records: (1) a full current-position × previous-position interaction covering all 3 × 3 transitions; (2) adjustment for original video route; (3) inclusion of a video random intercept in addition to participant random effects; (4) participant-clustered GEE estimation of the population-average association; and (5) restriction to participants with at least 80 valid choices in the phase. Participant-level random effects defined the primary model because repeated choices were nested within participants and the central inferential question concerned within-participant choice history. The video random-intercept specification was used to assess whether residual between-video heterogeneity altered the previous-choice estimate across the reused stimulus pool. These analyses evaluated the stability of the focal coefficient across alternative specifications. Coding and the definition of previous choice were unchanged. Supplementary Table S3 provides the full results. All effects are interpreted as conditional trial-level associations.

Because each Task 2 screen displayed the straight and cross-court probabilities for one current outcome dimension, we specified an explicit-information adjustment model. display_type used RALLY as the reference. The numerical information was defined as display_diff10 = (Pstraight − Pcross-court)/0.10, so one unit represented a 10-percentage-point signed difference in favor of straight. The model included display_type, display_diff10, and their interaction to allow the same numerical difference to have different directions or magnitudes for WIN, LOSS, and RALLY information. T2-Display included a participant random intercept and independent left- and right-position random slopes. T2-Full added both the fixed effect and participant random slope of the previous choice. Their likelihood-ratio test therefore evaluated the complete previous-choice extension, not the fixed coefficient alone. This analysis assessed whether the Task 2 context and previous-choice coefficients persisted after adjustment for the current displayed information. Consistent with its role as a descriptive, non-independent phase, Task 2 analyses focused on in-sample GLMM estimation and sensitivity analyses; rolling forward validation was reserved for the primary Task 1 analysis. Supplementary Table S4 gives the complete models and tests.

### 2.6. Computational Models and Rolling Forward Validation

In addition to the primary GLMM analysis, we conducted a secondary predictive model comparison for Task 1. We first compared the context model M1 with context-plus-previous-choice model M2 to test whether previous choice improved held-out prediction. M3 and M4 then separately tested whether outcome conditioning or contextual Q-learning added predictive information beyond M2. Final model inputs coded the current choice as straight = 1 and cross-court = 0 and the three opponent positions as center, left, and right states. Previous choice was coded *x_i_*_,*t*−1_ = +1 for straight on the immediately preceding numbered trial, −1 for cross-court, and 0 when that trial had no valid choice. Because route, key, and spatial response were perfectly collinear under the fixed mapping, *x_i_*_,*t*−1_ denotes the coded previous choice but cannot isolate a tactical route-choice effect. All models used a binary logistic choice rule that transformed the linear predictor *η_it_* into the probability of a straight choice.

*P*(*y_it_* = 1) = 1/[1 + exp(−*η_it_*)]

The context model (M1) contained only current position. Each participant had separate center, left, and right state biases *b_i_*_,*s*(*t*)_, for three participant-level parameters: *η_it_* = *b_i_*_,*s*(*t*)_. Model M2 added *κ_i_ x_i_*_,*t*−1_, for four participant-level parameters: *η_it_* = *b_i_*_,*s*(*t*)_ + *κ_i_ x_i_*_,*t*−1_. *κ_i_* describes the intertrial association between the previous and current choices; because choice is collinear with key and spatial response, it is not interpreted as a purely tactical route-choice parameter.

M2: *η_it_* = *b_i_*_,*s*(*t*)_ + *κ_i_ x_i_*_,*t*−1_

The outcome-dependent model (M3) used RALLY as the reference outcome and allowed the previous-choice coefficient to vary with the preceding outcome: *η_it_* = *b_i_*_,*s*(*t*)_ + *x_i_*_,*t*−1_[*κ*_RALLY,*i*_ + *δ*_WIN,*i*_ *I*(*r_i_*_,*t*−1_ = WIN) + *δ*_LOSS,*i*_ *I*(*r_i_*_,*t*−1_ = LOSS)]. Its six participant-level parameters comprised three state biases, one RALLY-reference previous-choice parameter, and two outcome-conditioned adjustments. M3 tested whether the previous-outcome category added predictive information beyond the parsimonious previous-choice model; it did not presume that a long-term value representation had formed.

M3: *η_it_* = *b_i_*_,*s*(*t*)_ + *x_i_*_,*t*−1_[*κ*_RALLY,*i*_ + *δ*_WIN,*i*_ *I*(WIN*_i_*_,*t*−1_) + *δ*_LOSS,*i*_ *I*(LOSS*_i_*_,*t*−1_)]

The contextual Q-learning model (M4) treated opponent positions as states *s* and straight and cross-court as actions *a* (Sutton & Barto, 2018). For every participant and validation fold, a 3 × 2 state–action value table was initialized at *Q*_1_(*s*,*a*) = 0. Feedback was coded WIN = +1, RALLY = 0, and LOSS = −1. The current trial was predicted from the existing Q values; after a valid choice and outcome, only the chosen action in the current state was updated. The values for the unchosen action and other states were unchanged. The value difference was *ΔQ_it_* = *Q_t_*(*s_t_*,Straight) − *Q_t_*(*s_t_*,Cross), which entered the choice rule through sensitivity *β_i_* while retaining current-position biases and previous choice. M4 had six participant-level parameters: three state biases, *κ_i_*, *α_i_*, and *β_i_*.

*δ_t_* = *r_t_* − *Q_t_*(*s_t_*,*a_t_*); *Q_t_*_+1_(*s_t_*,*a_t_*) = *Q_t_*(*s_t_*,*a_t_*) + *α_i_ δ_t_*; *Q_t_*_+1_(*s_t_*,*a* ≠ *a_t_*) = *Q_t_*(*s_t_*,*a*) M4: *η_it_* = *b_i_*_,*s*(*t*)_ + *κ_i_ x_i_*_,*t*−1_ + *β_i_*[*Q_t_*(*s_t_*,Straight) − *Q_t_*(*s_t_*,Cross)]

Parameters were estimated as participant-level maximum a posteriori (MAP) points with empirical-Bayes partial pooling, not by full Bayesian posterior sampling. Models were run in Python 3.12.13 with NumPy 2.5.2 and SciPy 1.18.1. Each participant’s objective was the training negative log-likelihood plus the Gaussian shrinkage penalty 0.5Σ*_j_*[(*θ_ij_* − *μ_j_*)/*σ_j_*]^2^. Group locations *μ* and scales *σ* were alternately updated from the mean and standard deviation of the current participant MAP estimates; no hyperprior was specified. The production rolling fits used a prespecified six-round empirical-Bayes alternating budget. Early stopping was defined as either a maximum absolute change in a group mean or SD below 1 × 10^−4^ or an absolute between-round change in total unpenalized training negative log-likelihood below 1 × 10^−5^. A subsequent convergence audit retained the same model definitions, objective functions, parameter bounds, optimizer settings, and validation folds while extending the maximum outer-loop budget to 50 rounds. None of the 12 model-by-fold fits met either stopping criterion within this extended budget. The six-round fits are therefore treated as fixed-budget predictive estimates, with the extended runs used as a convergence-sensitivity analysis. State biases, *κ*, and M3 *δ*_WIN_ and *δ*_LOSS_ were bounded on their original scales to [−6, 6]. In M4, *α_i_* = sigmoid(*α*_logit,i_), with *α*_logit_ ∈ [−6, 6] and, thus, *α* ∈ [0.00247, 0.99753]; *β_i_* = exp(log*β_i_*), with log*β* ∈ [−4, 4] and, thus, *β* ∈ [0.01832, 54.59815].

For each training cutoff, the three state biases were initialized from the smoothed empirical training logits logit[(successes + 0.5)/(*n* + 1)]. *κ* and outcome-conditioned *δ* terms began at 0; M4 began at *α* = 0.2 and *β* = 1. All parameters used one deterministic starting point, with no multiple starts or random restarts. As a post hoc optimization check, five additional admissible starting points per participant were evaluated at the round-50 terminal group hyperparameters; none improved the objective by more than 1 × 10^−6^. No full-data M4 *α*_logit_ or log*β* estimate was within 5% of its imposed parameter bounds (Supplementary Table S2A). Participant MAP estimates were calculated using scipy.optimize.minimize with L-BFGS-B. Rolling validation used maxiter = 120, ftol = 1 × 10^−9^, and gtol = 1 × 10^−6^. If the first optimization was unsuccessful, a single deterministic continuation began at that endpoint with maxiter = 240, ftol = 1 × 10^−9^, and gtol = 2 × 10^−6^. No optimization remained unsuccessful after retry. SEED = 20,260,825 was fixed for bootstrap and simulation procedures; deterministic parameter initialization did not use this seed.

Rolling forward validation used three fixed train–test folds: trials 1–45 predicted 46–60, trials 1–60 predicted 61–75, and trials 1–75 predicted 76–90. Each fold estimated participant parameters only from its training interval. Parameters remained fixed in the held-out interval. After trial *t* was predicted, the choice and outcome that had actually occurred could update the previous-choice or *Q* state needed for later trials; this was sequential one-step-ahead forecasting, with no refitting on future information. Missing choices contributed no choice likelihood, did not update Q, and broke previous choice on the next trial. A valid choice with a missing outcome retained its choice likelihood but did not update Q. All four models were evaluated on the same 2206 valid held-out Task 1 choices. Predictive performance is summarized by expected log predictive density (ELPD), ΔELPD, Brier score, and accuracy at a 0.50 threshold. Supplementary Table S5 reports complete log loss and mean log-score differences per held-out trial. Uncertainty in ΔELPD used participant clustering: paired trial-level log-score differences were summed within participant before estimating the interval across participants. An interval spanning 0 indicates no stable incremental predictive evidence under the present data and validation procedure.

For reproducibility, the final computational logic was independently reimplemented and rerun from the final trial-level model input. The common held-out sample remained 2206. The ELPD values for M1–M4 were −1073.614706, −1055.533560, −1053.785943, and −1051.581753, with a maximum absolute difference of 0.000440 from the existing results. Model ΔELPD values were also reproduced to within 0.001. This reproduction check confirmed the computational consistency of the implementation. Interpretation of the resulting model patterns remained tied to the assumptions embodied in the candidate structures. Supplementary Table S2 provides complete implementation and reproduction settings.

## 3. Results

### 3.1. Data Quality and Participant Characteristics

All 50 participants entered the primary analysis. Tasks 1 and 2 yielded 4402 and 4307 valid binary choices and 4295 and 4176 valid adjacent transitions, respectively. Among recorded rows, simultaneous missingness of choice and outcome accounted for 1.70% in Task 1 and 1.98% in Task 2, corresponding to 1.84% overall. Every candidate computational model used the same 2206 held-out Task 1 choices. Task 1 for P008 and Task 2 for P024 and P046 ended early because of program errors. Other structure and coding checks found no duplicate keys or illegal non-missing codes (Table 3). Restricting each phase to participants with at least 80 valid choices produced GLMM estimates close to those from the full sample, which indicates that the three incomplete records did not drive the Task 1 previous-choice association (Supplementary Table S3). Age and sex were summarized as sample characteristics, while the primary models focused on the prespecified task-level predictors.

### 3.2. Context Sensitivity of Route Choice: Association Between Current Position and Choice Probability

The observed choice proportions differed clearly across opponent positions. The values reported below are empirical proportions with participant-clustered 95% confidence intervals; model-based conditional associations are reported separately in the GLMM analyses. In Task 1, the probabilities of choosing straight at left, center, and right were 0.151 (95% CI [0.113, 0.191]), 0.570 (95% CI [0.531, 0.609]), and 0.856 (95% CI [0.818, 0.893]), respectively. The corresponding Task 2 probabilities were 0.114 (95% CI [0.078, 0.159]), 0.555 (95% CI [0.498, 0.605]), and 0.884 (95% CI [0.853, 0.912]) (Figure 3). Task 2 values are presented as descriptive estimates of the position–choice association in the fixed subsequent phase.

The GLMM likewise showed a significant association between current opponent position and route choice. In Task 1, relative to the center position, the odds of choosing straight were lower at left (OR = 0.087, 95% CI [0.059, 0.130], *p* < 0.001) and higher at right (OR = 6.297, 95% CI [4.400, 9.013], *p* < 0.001). Task 2 showed a directionally consistent descriptive separation. The overall Task 1 position × trial-progress interaction was nonsignificant, χ^2^(2) = 1.278, *p* = 0.528; there was no clear evidence that the position–choice association changed differentially over 90 trials (Table 4).

### 3.3. Primary GLMM Analysis: Previous Choice and Current Route Choice

On the same 4295 valid Task 1 transitions, both models retained identical participant random-effects structures. Adding the fixed effect of previous choice improved fit, LR χ^2^(1) = 36.453, *p* < 0.001. After adjustment for current position, position × trial progress, previous position, and position transition, a previous straight choice was associated with lower odds of choosing straight again (OR = 0.430, 95% CI [0.339, 0.546], *p* < 0.001). Under the fixed route–screen–key mapping, this pattern represents an alternation-type association in a composite response-history code spanning shot direction, spatial response, and keypress. This conditional previous-choice association is illustrated in Figure 4.

Rolling forward validation showed that adding previous choice to the context model increased total ELPD by 18.081 over the same 2206 held-out choices (SE = 6.533, 95% participant-clustered CI [5.277, 30.885]; clustered bootstrap CI [6.293, 30.987]). Brier score decreased from 0.160 to 0.156, and accuracy increased from 0.758 to 0.773, an absolute increase of 1.5 percentage points. Participant-level cumulative ΔELPD was positive for 33 of 50 participants and negative for 17. The mean participant-level gain was 0.362 (SD = 0.924), with a median of 0.374 (IQR = 1.199) and a range from −1.017 to 4.086. Previous-choice information improved the overall log predictive score, with variation in the magnitude and direction of the gain across participants. The absolute changes in probability error and classification accuracy remained small. When the most recent valid choice at the same opponent position was examined as a sensitivity variable, its gain over the context model was ΔELPD = 2.687 (95% CI [−0.231, 5.604]), with the interval spanning 0. The observed predictive increment was concentrated in the immediately preceding experimental response. Supplementary Table S5 provides the complete predictive metrics.

### 3.4. Robustness of the Previous-Choice Association and Descriptive Extension in Task 2

As a secondary descriptive analysis, the unadjusted Task 2 GLMM yielded a previous-choice OR of 0.544 (95% CI [0.421, 0.703]). After display_type, the signed between-route probability difference display_diff10, and their interaction were added, the estimate remained similar (β = −0.604, OR = 0.546, 95% CI [0.422, 0.707], *p* < 0.001).

The Task 1 previous-choice coefficient remained in the same direction after modeling all 3 × 3 position transitions, adjusting for original video route, adding a video random intercept, using participant-clustered GEE, and restricting the sample to participants with at least 80 valid choices. ORs ranged from 0.419 to 0.506, and every 95% CI was below 1. Task 2 point estimates were similar across several GLMM specifications, but participant-clustered GEE yielded OR = 0.856 (95% CI [0.632, 1.161], *p* = 0.318), with the interval including 1. Task 1 therefore provides the principal robustness evidence for the previous-response association within the experimental trial sequence. Task 2 remains a descriptive second phase with a fixed order, repeated stimuli, and explicit information. Supplementary Table S3 provides the complete results.

### 3.5. Secondary Analysis: Comparison of Candidate Predictive Models

These model comparisons assessed the incremental value of complex predictive structures relative to the main context–previous-choice baseline; they did not provide the primary statistical inference for the previous-choice effect. Among the 2206 common held-out choices, 2188 had a valid previous outcome. Stay probabilities after a previous WIN, RALLY, and LOSS were 0.461 (n = 1041), 0.440 (n = 782), and 0.392 (n = 365), respectively. The in-sample GLMM previous-choice × previous-outcome interaction was significant, χ^2^(2) = 22.008, *p* < 0.001. The WIN interaction had an OR = 2.097 (95% CI [1.457, 3.020]); the LOSS interaction had an OR = 0.856 (95% CI [0.531, 1.382]). These results indicate an outcome-conditioned association with the adjacent-trial stay/switch relation.

The context-plus-previous-choice model had a statistically detectable but small held-out gain over the context model. The outcome-dependent stay/switch model improved on the matched previous-choice model by only ΔELPD = 1.748 (95% CI [−0.402, 3.898]). Under the six-round production fit, the contextual Q-learning model yielded ELPD = −1051.582, corresponding to a ΔELPD of 3.952 relative to the context-plus-previous-choice model (95% CI [−8.734, 16.637]). Both intervals spanned 0. An extended outer-loop convergence audit produced the same qualitative pattern. After 50 rounds, the terminal M2–M1 comparison remained positive (ΔELPD = 19.378, 95% participant-clustered CI [2.160, 36.596]), whereas the terminal M4–M2 comparison remained uncertain (ΔELPD = 2.152, 95% CI [−10.609, 14.912]). None of the extended fits satisfied the prespecified fixed-point stopping criteria, so these terminal values are interpreted as sensitivity estimates. (Figure 5; Table 5). The ΔELPD intervals for both complex models spanned zero, which indicates uncertain incremental predictive value under the present validation procedure. The possible contribution of outcome-sensitive adjustment or value updating therefore remains an open mechanistic question.

## 4. Discussion

The primary evidence came from Task 1, the hidden-probability phase. Trial-level GLMMs showed a clear association between current opponent position and route choice. After adjustment for current and preceding spatial context, the immediately preceding choice remained associated with the current response. Previous-choice information also provided a modest improvement in the prediction of later held-out trials. Under the fixed route–screen–key mapping, this history term represents a composite sequential-response association spanning shot direction, spatial response, and keypress. The context-plus-previous-choice model also provided the most stable predictive account among the candidate structures, with no reliable incremental gain from the outcome-conditioned or contextual Q-learning extensions. Task 2 produced estimates in the same direction under the GLMM, while participant-clustered GEE provided weaker support; accordingly, this phase served as a secondary descriptive analysis. Overall, Task 1 provides evidence for context sensitivity and a previous-choice association within the experimental trial sequence.

The association between opponent position and route choice is consistent with the broader badminton literature showing that players make use of contextual information available before the terminal action. Badminton anticipation studies have shown that information extending beyond final stroke kinematics—including court configuration and preceding rally information—can contribute to action judgments (De Waelle et al., 2023; Lan et al., 2026). Naturalistic observations of expert badminton players likewise indicate that tactical decisions integrate information about the opponent, available court space, and the developing rally alongside stroke-related information (Macquet & Fleurance, 2007). The present findings extend this contextual perspective to repeated route selection: opponent position accounted for substantial variation in straight versus cross-court responses and provided the contextual baseline for evaluating recent choice history. No normative optimality was assigned to either route.

Previous choice contributed additional information in the primary Task 1 analysis. A previous straight response was associated with lower odds of another straight response on the immediately following trial, placing the coefficient in the direction of alternation. This association also contributed to prediction of later held-out choices. Across 2206 held-out observations, ΔELPD favored the context-plus-previous-choice model, while accuracy increased from 75.8% to 77.3% and the Brier score decreased from 0.160 to 0.156. The resulting predictive gain was detectable but modest. Its direction was retained across several robustness specifications. The association was specific to the immediately preceding recorded response; the same-position history analysis yielded no stable additional predictive gain.

The fixed response mapping defines the interpretive scope of the previous-choice association. Straight was consistently presented on the left side of the screen and selected with the F key; Cross-court was presented on the right and selected with the J key. Route identity, response location, and keypress therefore covaried across trials. The previous-choice coefficient consequently indexes persistence or alternation within this coupled route–spatial–motor response history. Separating the contributions of tactical route history, spatial response history, and keypress history will require independent manipulation of these mappings. The randomized sequence of independently recorded videos provides a second interpretive boundary: the observed association characterizes short-range dependence within the experimental choice sequence. Future studies can determine the sporting specificity of this pattern by independently counterbalancing route labels, screen locations, and response keys and by examining the same sequential structure in tasks that preserve rally continuity.

Task 2 addressed a narrower question concerning the robustness of the observed choice structure to the availability of explicit current outcome information. In the GLMM, the previous-choice estimate remained similar after adjustment for display type and the probability difference shown between routes, suggesting that the history term was not absorbed entirely by the numerical information presented at choice. This pattern was not reproduced by the participant-clustered GEE (OR = 0.856, 95% CI [0.632, 1.161]), indicating that the Task 2 estimate was sensitive to the estimation framework. Task 2 therefore informs the boundary conditions of the primary result. Context-related choice structure remained readily observable in the information-enriched phase, while evidence for the previous-choice association varied across estimation frameworks.

Stay probabilities differed descriptively after WIN, LOSS, and RALLY, and the in-sample interaction between previous choice and outcome was significant. Yet the held-out ΔELPD interval for the outcome-dependent model, relative to the matched previous-choice model, included 0. The incremental interval for contextual Q-learning, relative to the simpler previous-choice model, also included 0. Across the present candidate set and rolling-validation procedure, the context-plus-previous-choice baseline provided the most stable predictive account. The ΔELPD intervals for both the outcome-dependent and contextual Q-learning extensions included zero, and their accuracy and Brier scores differed little from the simpler model. These comparisons establish the relative predictive performance of the candidate structures, while the mechanistic contribution of outcome-sensitive adjustment or value updating remains open. The expert-derived feedback probabilities also differed in rating precision across outcome categories. Agreement was strongest for WIN and LOSS, whereas the RALLY estimate was lower and had a wider confidence interval. The lower precision for RALLY may reflect the broader range of tactical states encompassed by continued play, compared with the more clearly bounded terminal outcomes of WIN and LOSS. This broader category may have allowed greater variation in expert probability judgments. The specific sources of this variation were not measured directly. In the sensitivity analysis, however, alternative probability construction changed no normalized cell by more than 2 percentage points, indicating that the implemented feedback matrix was stable across inclusion rules. Analyses involving the detailed outcome structure, particularly RALLY and the outcome-sensitive candidate models, therefore carry greater uncertainty than the primary context and previous-choice estimates.

The computational model comparison also showed sensitivity to the empirical-Bayes outer-loop iteration budget. The six-round production results were reproduced exactly, while the prespecified fixed-point stopping criteria were not reached during an extended 50-round convergence audit. Absolute ELPD values and participant-level learning parameters therefore warrant cautious interpretation. The central comparative pattern remained consistent in the extended analysis: previous-choice information retained a predictive advantage over context alone, whereas the contextual Q-learning extension showed no stable incremental advantage over the simpler baseline.

The sample consisted of badminton players aged 13 to 15 years who had completed 5 to 7 years of systematic training and were registered as district representative-team athletes. The study included no comparison with another age group or with adults, and it did not stratify competitive level using formal athlete grades or objective competition results. The sample was also sex-imbalanced (36 boys and 14 girls), providing limited information for estimating sex-related differences in context sensitivity or previous-choice associations. Sex was therefore summarized descriptively in the present analysis. The findings are consequently limited to the behavioral structure observed in this particular adolescent sport sample. Research using broader age ranges, more balanced samples, longitudinal designs, or explicit expertise strata is needed to examine the potential roles of developmental age, sex, training history, and competitive level.

Several methodological limitations affect the interpretation of the results. A major limitation concerns the ecological representativeness of the response setting. The video stimuli preserved badminton-specific spatial and opponent information, and the keyboard response provided a standardized measure of route selection across repeated trials. The resulting paradigm captures response selection under controlled visual conditions, whereas on-court badminton integrates information sampling, movement preparation, footwork initiation, stroke execution, and online adjustment within a continuously evolving perception–action process. Three participant-task records ended early because of program errors. Restricting the analysis to records with at least 80 valid choices produced a similar Task 1 estimate, although these program-related interruptions reduced data completeness. Visual search and motor preparation are therefore important targets for subsequent work. Participants in the present study viewed a fixed two-dimensional display, with gaze behavior falling outside the measured variables. Keyboard responses likewise impose substantially lower sport-specific motor demands than court movement and stroke execution. Research in sport cognition has shown that response modality and stimulus representativeness can influence the validity and temporal characteristics of decision tasks, and sport-specific motor responses may engage additional preparation processes that are absent from simple button presses (Pinder et al., 2011; Musculus et al., 2022; Höner et al., 2023). Future studies could examine the present choice-history pattern across progressively more representative settings, including counterbalanced manual responses, badminton-specific stepping or racket responses, eye-tracking, and interactive or on-court tasks that preserve the evolving perception–action cycle. This progression would clarify how sequential associations observed during controlled response selection extend to embodied tactical behavior. The original stimulus records also did not retain the exact frame-wise interval between occlusion and contact, which limited precise reproduction of the stimulus timing. Finally, this study included no training intervention, transfer test, or external measure of match performance. It describes the structure of repeated sport-specific choices but does not establish whether that structure is trainable or related to competitive performance. Future research could combine sport-specific motor responses and real-time interaction with longitudinal or training designs to examine the stability, plasticity, and performance relevance of these trial-to-trial choice associations.

## 5. Conclusions

Among adolescent badminton players completing repeated video-based shot-direction decisions, opponent position was strongly associated with straight versus cross-court responses. The immediately preceding recorded response was also associated with the current response and provided a modest out-of-sample predictive increment beyond the contextual baseline. This predictive increment was concentrated in the immediately preceding experimental response, with no stable additional gain from the most recent choice made at the same opponent position. Under the fixed route–screen–key mapping, the history term indexes a composite sequential-response signal spanning tactical, spatial, and motor-response components. Outcome-sensitive and contextual Q-learning structures provided no stable incremental predictive advantage under the present design and validation procedure.

## Figures and Tables

**Figure 1:**
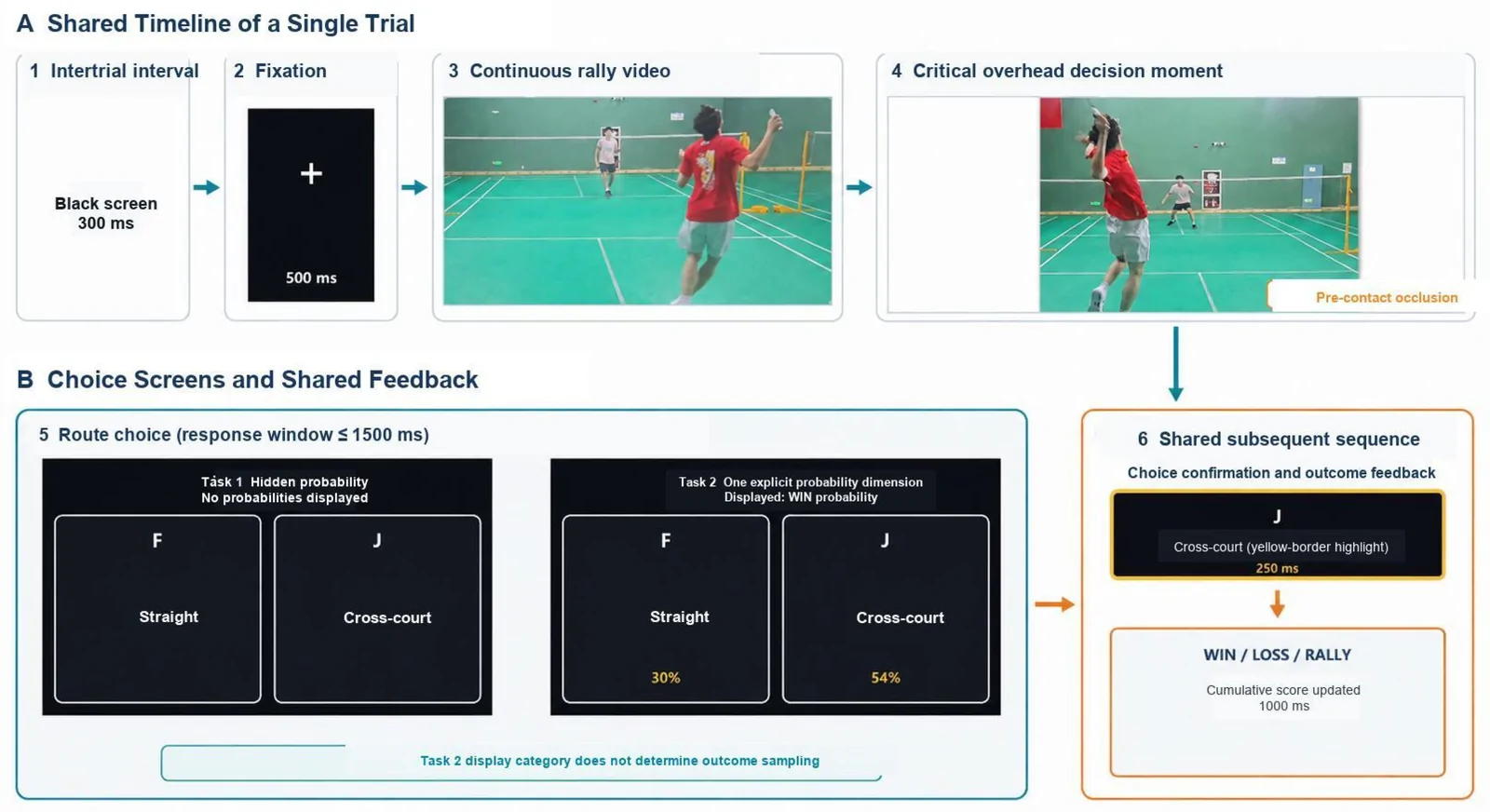
Timeline of a single trial and choice screens in the hidden-probability phase (Task 1) and explicit-probability phase (Task 2). Note. The shared sequence comprised a 300 ms intertrial interval, 500 ms fixation, a video containing the preceding rally, the critical pre-contact decision moment, route choice, 250 ms choice highlighting, and 1000 ms outcome feedback. No additional final frame hold followed the video. Task 1 displayed no probabilities. In each Task 2 trial, only one outcome dimension—WIN, LOSS, or RALLY—was displayed, with the corresponding probabilities for the two routes. The illustrative values of 30% and 54% are the WIN probabilities for straight and cross-court choices in the left-position context, respectively (Table 1). The displayed outcome type did not determine backend outcome sampling.

**Figure 2:**
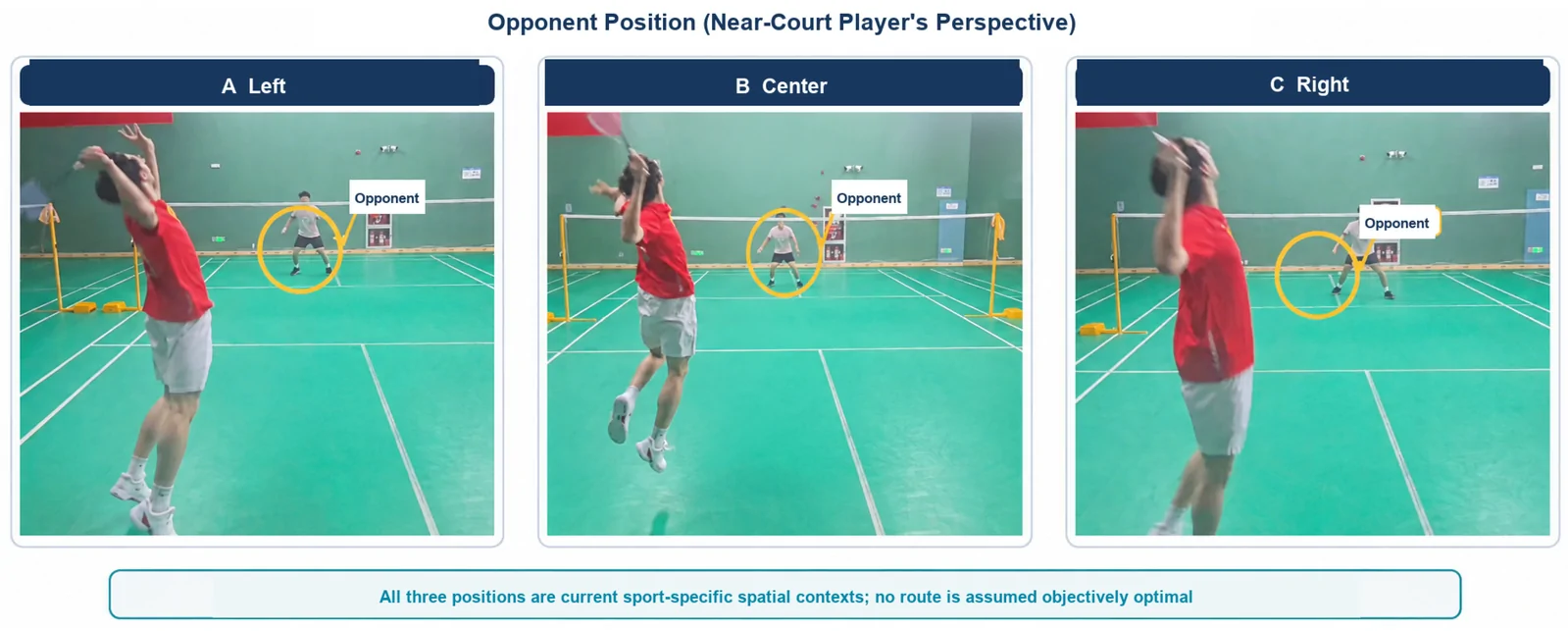
Opponent position and sport-specific context. Note. Left, center, and right are defined from the near-court player’s perspective. Yellow circles identify the far-court opponent. The three positions constituted the current trial’s sport-specific spatial context.

**Figure 3:**
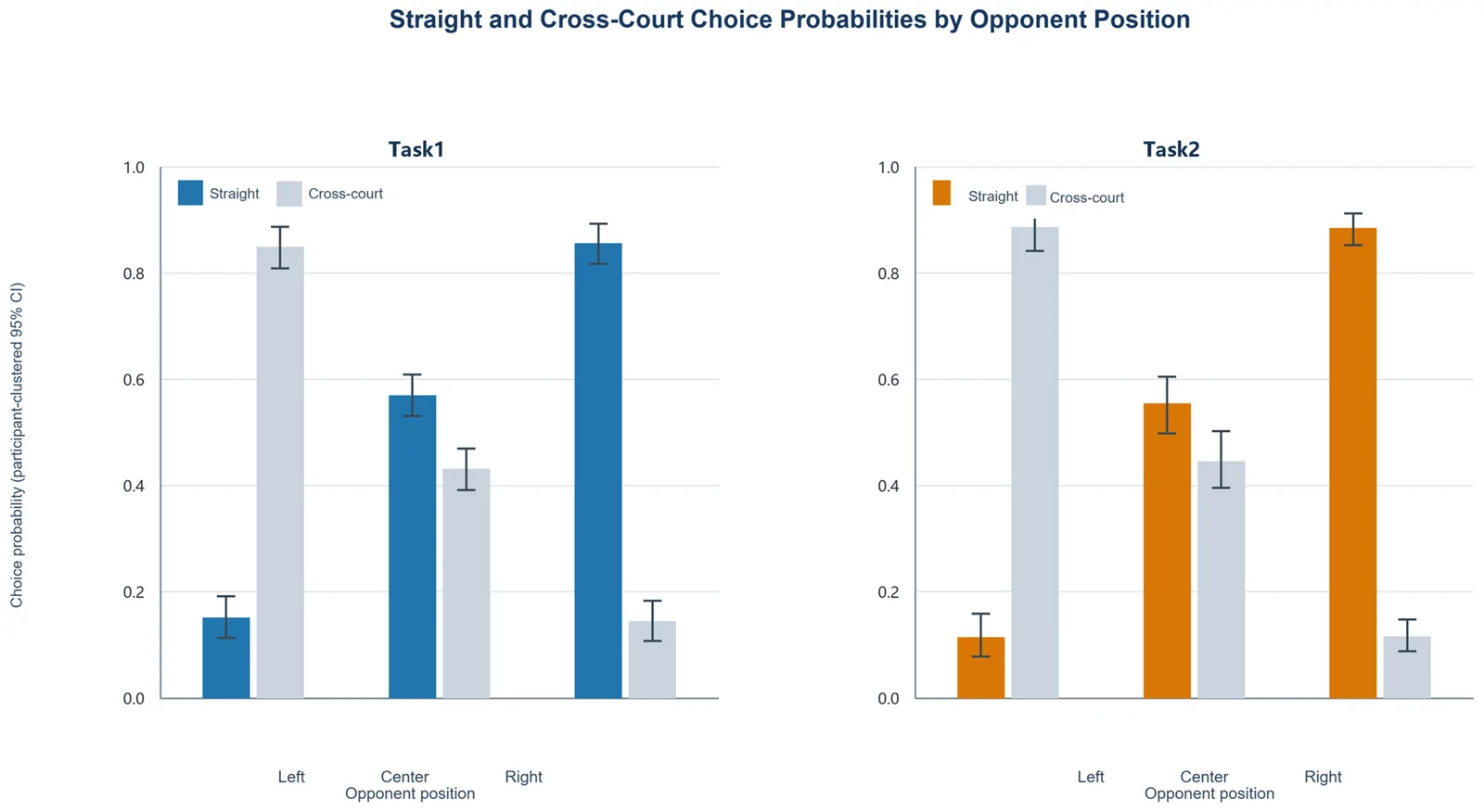
Probabilities of straight and cross-court choices at each opponent position. Note. Error bars are participant-clustered 95% intervals. Task 2 was the descriptive second phase under a fixed order with repeated stimuli.

**Figure 4:**
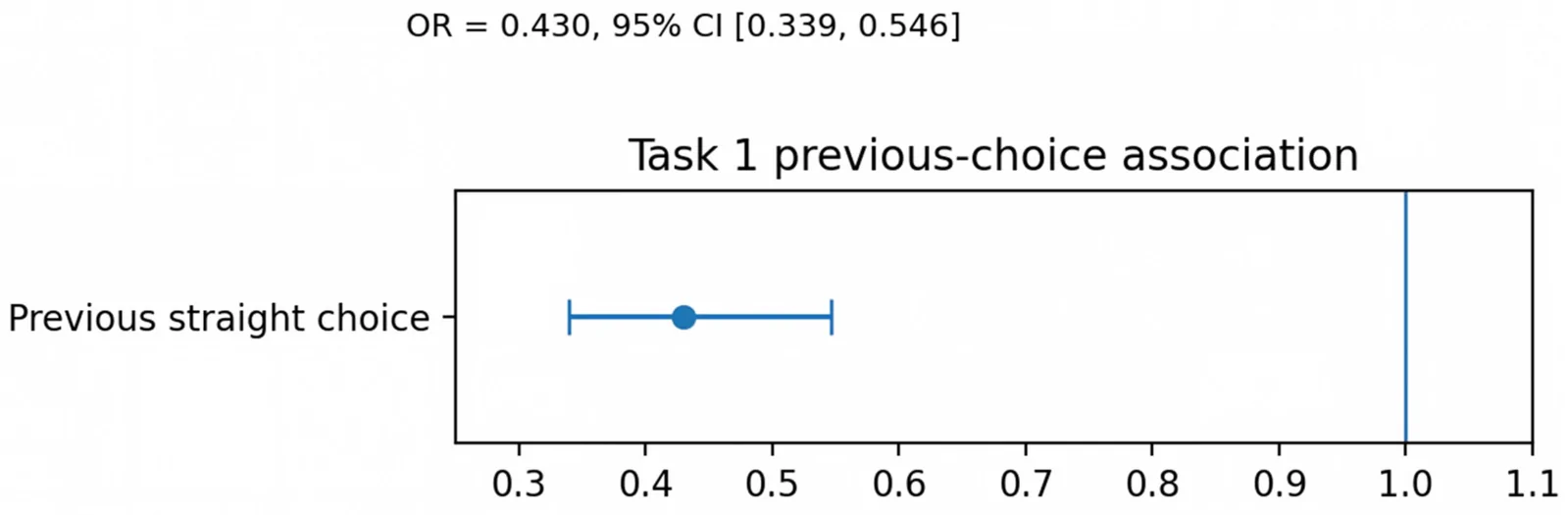
Conditional association between previous choice and current route choice in Task 1. Note. The estimate is derived from the primary Task 1 GLMM after adjustment for current position, position × trial progress, previous position, and position transition. An OR below 1 indicates an alternation-type association within the fixed route–spatial–key response mapping. The coefficient therefore represents a composite response-history signal spanning shot direction, spatial response, and keypress. Task 2 estimates are reported separately in Section 3.4 and Supplementary Tables S3 and S4.

**Figure 5:**
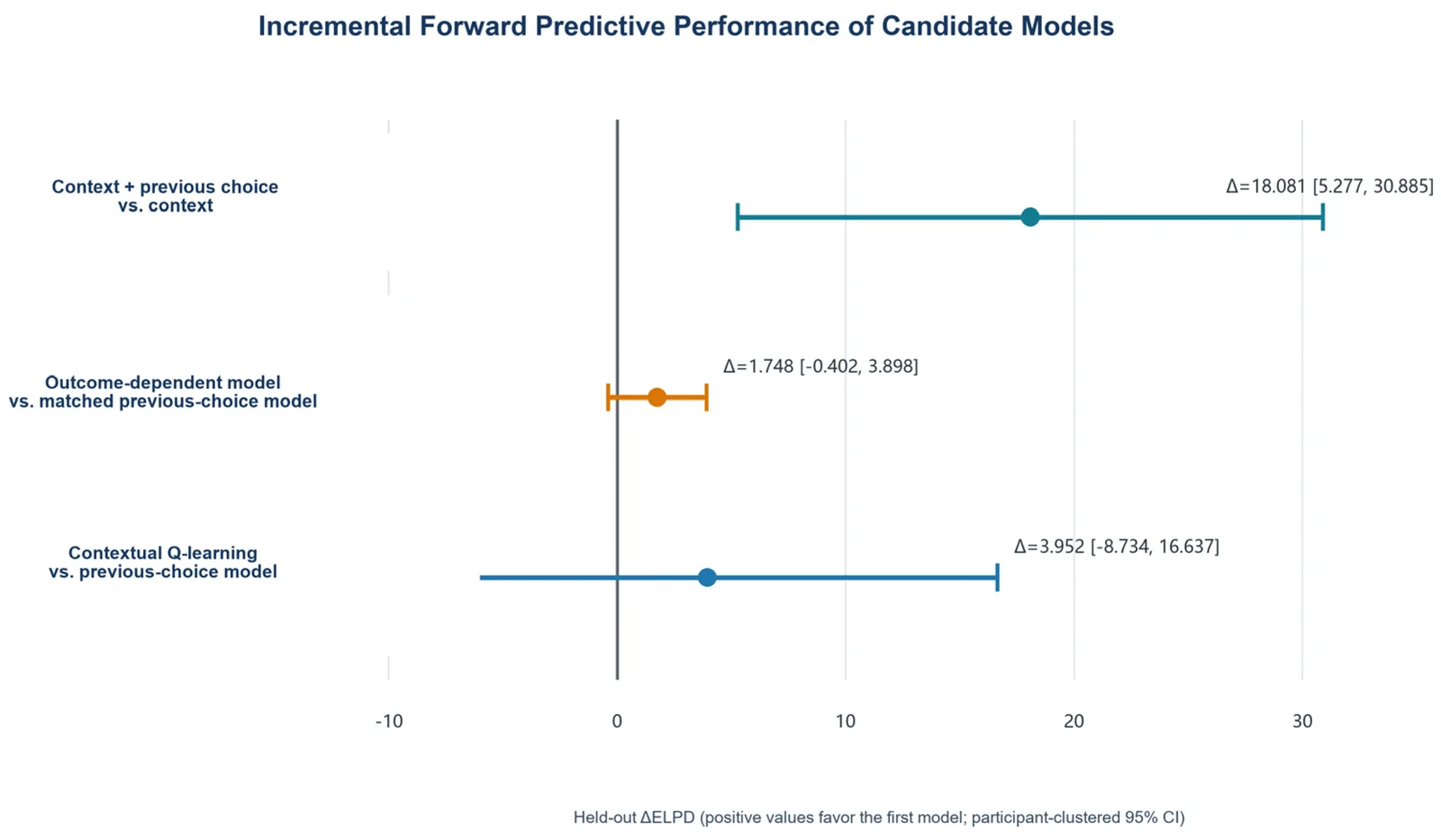
Incremental forward predictive performance of the candidate models. Note. A ΔELPD interval spanning 0 indicates no stable incremental predictive evidence in the present validation data. It does not establish model equivalence or the absence of a complex learning process.

**Table 1:** Expert-derived simulated outcome probabilities used by the program. Note. Probabilities are ordered as WIN, LOSS, and RALLY; straight/cross-court denotes the participant’s keypress choice. Each row is based on valid expert judgments in the corresponding condition and sums to 1 after averaging, normalization, and integerization.

Opponent Position	Participant Choice	WIN	LOSS	RALLY
Left	Straight	0.30	0.25	0.45
Left	Cross-court	0.54	0.17	0.29
Center	Straight	0.38	0.15	0.47
Center	Cross-court	0.38	0.17	0.45
Right	Straight	0.59	0.13	0.28
Right	Cross-court	0.22	0.38	0.40

**Table 2:** Agreement in the expert probability ratings. Note. ICC(A,k) used a two-way random-effects, absolute-agreement, average-measures formulation. Confidence intervals are based on the 20 complete experts who provided valid ratings in all six position × route conditions.

Outcome Dimension	Complete Experts N	ICC(A,k)	95% CI
WIN	20	0.918	[0.79, 0.99]
LOSS	20	0.894	[0.73, 0.98]
RALLY	20	0.776	[0.47, 0.96]

**Table 3:** Participant characteristics and data completeness. Note. The primary analysis included all 50 participants. Age and training duration were rechecked against on-site registration records. All incomplete tasks ended because of program errors.

Measure	Result
Sample size	50
Sex	36 boys; 14 girls
Age, years	13.70 ± 0.71; range 13–15
Age distribution	Age 13: 22; age 14: 21; age 15: 7
Training duration, years	5.82 ± 0.80; range 5–7
Training-duration distribution	5 years: 21; 6 years: 17; 7 years: 12
Formal athlete technical grade	None: 50 (100%)
Recorded rows	Task 1: 4478; Task 2: 4394; total: 8872
Valid binary choices	Task 1: 4402; Task 2: 4307
Valid adjacent transitions	Task 1: 4295; Task 2: 4176
Choice and outcome both missing	163/8872 (1.84%); Task 1: 76/4478 (1.70%); Task 2: 87/4394 (1.98%)
Incomplete participant–task records	P008 Task 1: 68/90; P024 Task 2: 39/90; P046 Task 2: 35/90 (all program errors)
Corresponding valid binary choices	P008 Task 1: 64; P024 Task 2: 38; P046 Task 2: 32
Sample with ≥80 valid choices	Task 1: n = 49; Task 2: n = 48
Held-out computational-model choices	2206 (50 participants; identical for all models)

**Table 4:** Primary generalized linear mixed-effects model results for Task 1. Note. Current choice was coded straight = 1 and cross-court = 0; center was the contextual reference. Position effects are conditional statistical associations. The table summarizes the focal fixed effects from the primary Task 1 models; complete fixed-effect estimates for all adjustment variables are reported in Supplementary Table S1A. Both sides of the Task 1 previous-choice likelihood-ratio test used identical random-effects structures, with the 1 df difference arising from the fixed previous-choice term. Task 2 results are reported in Section 3.4 and Supplementary Tables S3 and S4.

Task	Fixed Effect	OR	95% CI	*p*	n
Task 1	Left vs. center	0.087	[0.059, 0.130]	<0.001	4402
Task 1	Right vs. center	6.297	[4.400, 9.013]	<0.001	4402
Task 1	Left × trial progress	1.285	[0.679, 2.431]	0.442	4402
Task 1	Right × trial progress	1.414	[0.742, 2.696]	0.293	4402
Task 1	Previous choice	0.430	[0.339, 0.546]	<0.001	4295

**Table 5:** Core forward predictive comparisons among Task 1 candidate models. Note. All models were evaluated on the same 2206 held-out choices. Higher ELPD and lower Brier scores indicate better prediction. ΔELPD 95% CIs use participant clustering. Complete log loss and mean log-score differences per held-out trial appear in Supplementary Table S5. An extended 50-round outer-loop sensitivity analysis did not reach the prespecified fixed-point stopping criteria; the corresponding terminal model comparisons are reported in the Supplementary Materials.

Candidate Model	ELPD	Brier	Accuracy	Comparator	ΔELPD [95% CI]
Context	−1073.615	0.160	0.758	Reference	—
Context + previous choice	−1055.534	0.156	0.773	vs. context	18.081 [5.277, 30.885]
Outcome-dependent stay/switch	−1053.786	0.156	0.772	vs. matched previous-choice model	1.748 [−0.402, 3.898]
Contextual Q-learning	−1051.582	0.156	0.775	vs. previous-choice model	3.952 [−8.734, 16.637]

## Data Availability

Because this study involved minors, the sharing of trial-level data is constrained by informed-consent, ethics-approval, and privacy-protection requirements. De-identified data may be obtained from the corresponding author upon reasonable request and subject to the applicable requirements. Code used for the primary analyses may be obtained through the same procedure.

## Supplementary Materials

The following supporting information can be downloaded at: https://www.mdpi.com/article/10.3390/bs16101721/s1, Table S1: Fixed- and random-effects specifications and fitting diagnostics for the reported GLMMs; Table S1A: Complete fixed-effect reporting structure for the primary Task 1 GLMMs; Table S2: Complete implementation and reproduction settings for the Task 1 candidate computational models; Table S2A: Extended empirical-Bayes outer-loop convergence and parameter-stability audit; Table S3: Complete robustness analyses of the previous-choice coefficient; Table S4: Task 2 explicit-information adjustment models and related tests; Table S5: Complete forward predictive metrics for the Task 1 candidate models.
