# Supplementary material for: Context Sensitivity and Previous-Choice Associations in Repeated Shot-Direction Decisions Among Adolescent Badminton Players
Source: Behav Sci (Basel). 2026 Sep 22;16(10):1721. doi: 10.3390/bs16101721 (PMC13701375; doi:10.3390/bs16101721)
Supplement: Supplementary file 1 [file behavsci-16-01721-s001.zip › behavsci-4563558-supplementary.pdf]

Supplementary Materials

Table S1. Fixed- and random-effects specifications and fitting diagnostics for the reported GLMMs

Task	Model/purpose	Fixed-effect formula	Initial random effects	Final random effects	Simplified	Convergence diagnostic	Singular fit	Optimization
Task 1	Current position/context	y ~ position	R-pos	R-pos	No; first candidate retained	code = 0; max|grad| = 5.67×10^{-5}	No	bobyqa; maxfun = 200,000; nAGQ = 1
Task 1	Context × trial progress	y ~ position * trial_c	R-pos	R-pos	No; first candidate retained	code = 0; max|grad| = 5.64×10^{-5}	No	bobyqa; maxfun = 200,000; nAGQ = 1
Task 1	Previous-choice test: null	y ~ position * trial_c + previous_position_f + same_pos	R-hist	R-hist	No; first candidate retained	code = 0; max|grad| = 6.84×10^{-5}	No	bobyqa; maxfun = 200,000; nAGQ = 1
Task 1	Primary previous-choice model	y ~ position * trial_c + previous_position_f + same_pos + previous_choice	R-hist	R-hist	No; first candidate retained	code = 0; max|grad| = 1.01×10^{-4}	No	bobyqa; maxfun = 200,000; nAGQ = 1
Task 1	Outcome main-effect extension	y ~ position * trial_c + previous_position_f + same_pos + prev_choice_c + previous_outcome_f	R-hist-c	R-hist-c	No; first candidate retained	code = 0; max|grad| = 9.13×10^{-5}	No	bobyqa; maxfun = 200,000; nAGQ = 1
Task 1	Outcome-conditioned previous-choice extension	y ~ position * trial_c + previous_position_f + same_pos + prev_choice_c * previous_outcome_f	R-hist-c	R-hist-c	No; first candidate retained	code = 0; max|grad| = 1.52×10^{-4}	No	bobyqa; maxfun = 200,000; nAGQ = 1
Task 1	Previous choice + video random intercept	choice_straight ~ position * progress_c + previous_position + same_position + previous_choice	R-hist+V	R-hist+V	No; directly specified	max|grad| = 9.06×10^{-5}; warnings empty	No	bobyqa; maxfun = 300,000; nAGQ = 1
Task 2	Unadjusted previous-choice model	choice_straight ~ position * progress_c + previous_position + same_position + previous_choice	R-hist	R-hist	No; directly specified	max|grad| = 5.63×10^{-5}; warnings empty	No	bobyqa; maxfun = 300,000; nAGQ = 1
Task 2	Unadjusted previous choice + video random intercept	choice_straight ~ position * progress_c + previous_position + same_position + previous_choice	R-hist+V	R-hist+V	No; directly specified	max|grad| = 9.63×10^{-5}; warnings empty	No	bobyqa; maxfun = 300,000; nAGQ = 1

Task	Model/purpose	Fixed-effect formula	Initial random effects	Final random effects	Simplified	Convergence diagnostic	Singular fit	Optimization
Task 2	T2-Display	choice_straight ~ position * progress_c + previous_position + same_position + display_type * display_diff10	R-pos	R-pos	No; directly specified	max|grad| = 2.55 × 10⁻⁴; warnings empty	No	bobyqa; maxfun = 300,000; nAGQ = 1
Task 2	T2-Full	choice_straight ~ position * progress_c + previous_position + same_position + display_type * display_diff10 + previous_choice	R-hist	R-hist	No; directly specified	max|grad| = 1.94 × 10⁻⁴; warnings empty	No	bobyqa; maxfun = 300,000; nAGQ = 1
Task 2	T2-Full + video random intercept	choice_straight ~ position * progress_c + previous_position + same_position + display_type * display_diff10 + previous_choice	R-hist+V	R-hist+V	No; directly specified	max|grad| = 1.83 × 10⁻⁴; warnings empty	No	bobyqa; maxfun = 300,000; nAGQ = 1
Task 2	Same-position secondary sensitivity	y ~ position * trial_c + prev_choice_c * previous_outcome_f	R-hist-c	R-pos	Yes; previous-choice random slope removed	final code = 0; max|grad| = 2.45 × 10⁻⁵	Initial: yes; final: no	bobyqa; maxfun = 200,000; nAGQ = 1

Note. R-pos = (1 + pos_left + pos_right || participant); R-hist = (1 + pos_left + pos_right + previous_choice || participant); R-hist-c = (1 + pos_left + pos_right + prev_choice_c || participant); V = +(1 | video). Double vertical bars (||) indicate independent participant random intercepts and slopes, with no estimated random-effect correlations. For the primary Task 1 previous-choice likelihood-ratio comparison, previous choice was coded cross-court = 0 and straight = 1 in both the fixed effect and participant random slope. Singular fits were assessed uniformly with lme4::isSingular(model, tol = 1×10^{-4}).

Table S1A. Complete fixed-effect reporting structure for the primary Task 1 GLMMs

Model	Fixed effect	OR	Wald 95% CI	p	n
Current-position/context model	Intercept (center)	1.341	[1.137, 1.581]	<.001	4402
Current-position/context model	Left vs. center	0.087	[0.059, 0.130]	<.001	4402
Current-position/context model	Right vs. center	6.307	[4.406, 9.030]	<.001	4402
Context × trial-progress model	Intercept (center at centered trial progress)	1.342	[1.138, 1.584]	<.001	4402
Context × trial-progress model	Left vs. center	0.087	[0.059, 0.130]	<.001	4402
Context × trial-progress model	Right vs. center	6.297	[4.400, 9.013]	<.001	4402
Context × trial-progress model	Trial progress	0.748	[0.518, 1.081]	.122	4402
Context × trial-progress model	Left × trial progress	1.285	[0.679, 2.431]	.442	4402
Context × trial-progress model	Right × trial progress	1.414	[0.742, 2.696]	.293	4402
Primary previous-choice model	Intercept	2.155	[1.665, 2.790]	<.001	4295
Primary previous-choice model	Current position: Left vs. center	0.083	[0.055, 0.125]	<.001	4295
Primary previous-choice model	Current position: Right vs. center	6.514	[4.479, 9.475]	<.001	4295
Primary previous-choice model	Trial progress	0.775	[0.523, 1.147]	.202	4295

Model	Fixed effect	OR	Wald 95% CI	p	n
Primary previous-choice model	Left × trial progress	1.093	[0.560, 2.130]	.795	4295
Primary previous-choice model	Right × trial progress	1.240	[0.625, 2.459]	.539	4295
Primary previous-choice model	Previous position: Left vs. center	0.989	[0.798, 1.226]	.920	4295
Primary previous-choice model	Previous position: Right vs. center	1.117	[0.911, 1.368]	.287	4295
Primary previous-choice model	Same-position transition	0.831	[0.696, 0.992]	.041	4295
Primary previous-choice model	Previous choice	0.430	[0.339, 0.546]	<.001	4295

Note. ORs, Wald 95% CIs, and p values are reported for all fixed-effect terms in the primary Task 1 GLMMs. Center served as the reference level for current and previous opponent position. Trial progress was centered at 0.5. Previous choice was coded cross-court = 0 and straight = 1. The primary previous-choice model included independent participant random slopes for left position, right position, and previous choice. The population-level contribution of previous choice was additionally evaluated by a likelihood-ratio comparison of models with identical random-effects structures (LR $\chi^2(1) = 36.453$, $p < .001$).

Table S2. Complete implementation and reproduction settings for the Task 1 candidate computational models

Element	Reproduction implementation
Environment and randomness	Python 3.12.13; NumPy 2.5.2; SciPy 1.18.1. SEED = 20260825 fixed for bootstrap and simulation. M1–M4 production fits used deterministic empirical initial values with no random initialization or random restarts; a post-hoc multiple-start diagnostic is reported in Table S2A.
Choice and state coding	straight = 1; cross = 0; state = center/left/right; previous choice +1 = straight and −1 = cross, or 0 when missing.
M1 / M2 / M3 / M4	M1 = 3 participant state biases; M2 = M1 + κ previous choice; M3 = outcome-conditioned previous choice with RALLY as reference; M4 = M2 + β·ΔQ, including α and β.
Q-learning	All Q values initialized at 0; WIN = +1, RALLY = 0, LOSS = −1; only chosen-action Q updated; unchosen actions and other states unchanged.
Bounds and transformations	b, κ, δ ∈ [−6, 6]; α = sigmoid(α_logit), α_logit ∈ [−6, 6]; β = exp(logβ), logβ ∈ [−4, 4].
Initialization	State bias = smoothed empirical training logit[(successes + 0.5)/(n + 1)]; κ/δ = 0; α = 0.2; β = 1. The production analysis used one deterministic starting point. The post-hoc round-50 diagnostic used the deterministic start plus five additional admissible starting points per participant.
Partial pooling and stopping	Empirical-Bayes participant MAP; Gaussian shrinkage to current group mean/SD; no hyperprior or MCMC. Production rolling fits used a prespecified maximum of 6 alternating rounds; early stopping required either a maximum absolute group mean/SD change $<1 \times 10^{-4}$ or a between-round total unpenalized training NLL change $<1 \times 10^{-5}$. All 12 model-by-fold fits reached the 6-round budget without meeting either criterion. A post-hoc audit retained the same model definitions, objective, bounds, optimizer settings, and validation folds and extended the outer-loop maximum to 50 rounds; none met the stopping criteria by round 50. Descriptive full-data production fits allowed 10 rounds.
Optimization	scipy.optimize.minimize, L-BFGS-B. Rolling: maxiter = 120, ftol = 1×10^{-9}, gtol = 1×10^{-6}; one deterministic continuation from endpoint if unsuccessful (maxiter = 240, ftol = 1×10^{-9}, gtol = 2×10^{-6}); 0 failures after retry. Full-data descriptive fits used maxiter = 180 and retry = 360.
Forward validation	1–45→46–60; 1–60→61–75; 1–75→76–90. Parameters fixed in test intervals; observed choices/outcomes updated history/Q sequentially. Common held-out N = 2206.

Table S2A. Extended empirical-Bayes outer-loop convergence and parameter-stability audit

Candidate model	6-round ELPD	Round-50 terminal ELPD	Brier	Accuracy	Log loss
Context	−1073.615	−1103.108	0.163	0.758	0.500
Context + previous choice	−1055.534	−1083.730	0.160	0.773	0.491
Outcome-dependent stay/switch	−1053.786	−1080.906	0.159	0.767	0.490
Contextual Q-learning	−1051.582	−1081.578	0.159	0.774	0.490

Note. Round-50 values are terminal sensitivity estimates; none of the 12 rolling model-by-fold fits met the prespecified fixed-point stopping criteria within 50 rounds. At round 50, ΔELPD was 19.378 (95% participant-clustered CI [2.160, 36.596]) for M2 versus M1 and 2.152 (95% CI [−10.609, 14.912]) for M4 versus M2. In the full-data M4 audit, no α_logit or logβ estimate was within 1%, 2.5%, or 5% of its parameter bounds. Participant-level rankings were broadly preserved from round 6 to round 50 (α: r = 0.955; β: r = 0.993), although parameter values continued to change. At the round-50 terminal group hyperparameters, five additional admissible starting points per participant produced no objective improvement greater than 1×10^{-6} over the deterministic solution.

Table S3. Complete robustness analyses of the previous-choice coefficient

Task	Analysis	N	Adjacent transitions n	Previous-choice OR [95% CI]	p
Task 1	S0 Primary GLMM	50	4295	0.430 [0.339, 0.546]	<.001
Task 1	S1 Full position transition	50	4295	0.436 [0.343, 0.554]	<.001
Task 1	S2 + original video route	50	4295	0.430 [0.339, 0.546]	<.001
Task 1	S3 + video random intercept	50	4295	0.421 [0.330, 0.537]	<.001
Task 1	S4 Participant-clustered GEE	50	4295	0.506 [0.386, 0.664]	<.001
Task 1	S5 ≥80 valid choices	49	4235	0.419 [0.328, 0.535]	<.001
Task 2	S0 Primary GLMM (unadjusted)	50	4176	0.544 [0.421, 0.703]	<.001
Task 2	S1 Full position transition	50	4176	0.537 [0.415, 0.696]	<.001
Task 2	S2 + original video route	50	4176	0.543 [0.420, 0.702]	<.001
Task 2	S3 + video random intercept	50	4176	0.526 [0.405, 0.682]	<.001
Task 2	S4 Participant-clustered GEE	50	4176	0.856 [0.632, 1.161]	.318
Task 2	S5 ≥80 valid choices	48	4110	0.546 [0.419, 0.710]	<.001

Note. OR < 1 denotes an alternation direction under the fixed route–spatial–key mapping. S5 tests whether the three incomplete records caused by program termination drove the result. The Task 2 GEE interval spans 1; the main text therefore treats Task 2 as a descriptive secondary phase and does not use it as independent robust support for the central previous-choice association.

Table S4. Task 2 explicit-information adjustment models and related tests

Analysis/test	Estimate or statistic	95% CI	p	Interpretive boundary
Previous choice without explicit-information adjustment	OR = 0.544	[0.421, 0.703]	<.001	Unadjusted Task 2 previous-choice estimate
Previous choice adjusted for explicit information	OR = 0.546	[0.422, 0.707]	<.001	Change in |β| <1%
Explicit adjustment + video random intercept	OR = 0.527	[0.406, 0.684]	<.001	video SD = 0.375; converged and nonsingular
Explicit-information block vs. Base	LR $\chi^2(5) = 3.581$	—	.611	No clear incremental fit
display type (probability difference = 0)	Wald $\chi^2(2) = 0.080$	—	.961	Joint test
Any displayed-probability-difference slope	Wald $\chi^2(3) = 1.785$	—	.618	Joint test
display type × probability difference	LR $\chi^2(2) = 1.014$	—	.602	Interaction-block test
Complete previous-choice extension vs. T2-Display	LR $\chi^2(2) = 33.009$	—	<.001	Adds both previous-choice fixed effect and participant previous-choice random slope

Note. The first three rows report previous-choice coefficients; the remaining rows report model-block or joint tests. The 2 df in the complete previous-choice extension comprise one fixed previous-choice effect and one variance parameter for the participant previous-choice random slope. The LRT evaluates overall fit of T2-Full relative to T2-Display; the previous-choice fixed effect is reported using its OR, Wald 95% CI, and p value.

Table S5. Complete forward predictive metrics for the Task 1 candidate models

Candidate model	ELPD	log loss	Brier	Accuracy	Comparator	ΔELPD	Mean Δlog-score/trial
Context	−1073.615	0.487	0.160	0.758	Reference	—	—
Context + previous choice	−1055.534	0.478	0.156	0.773	vs. context	18.081	0.0082
Outcome-dependen t stay/switch	−1053.786	0.478	0.156	0.772	vs. matched previous-choice model	1.748	0.0008
Contextual Q-learning	−1051.582	0.477	0.156	0.775	vs. previous-choice model	3.952	0.0018

Note. All models used the same 2206 held-out choices. Higher ELPD and lower log loss and Brier scores indicate better prediction. ΔELPD is the cumulative difference in log predictive score across held-out trials; mean Δlog-score/trial is ΔELPD divided by 2206. In the primary context-plus-previous-choice comparison, accuracy increased by 1.5 percentage points and Brier score decreased by 0.004. These values are from the prespecified six-round production fits. An extended 50-round outer-loop audit did not meet the fixed-point stopping criteria; the corresponding terminal sensitivity results and parameter diagnostics are reported in Table S2A.
